# Supplementary material for: Disorder affects judgements about a neighbourhood: police presence does not
Source: PeerJ. 2014 Mar 4;2:e287. doi: 10.7717/peerj.287 (PMC3961159; doi:10.7717/peerj.287)
Supplement: Appendix S1 [file peerj-02-287-s001.docx]

**Appendix 1**

Vignette of the deprived neighbourhood with police presence in italics:

You will now read a short description of a city neighbourhood.

Please read the description carefully.

Imagine you are walking through this neighbourhood.

Walking along the high street you pass a church and some stores. The stores include a supermarket called ‘Save-a-lot’, a liquor store with the shutters down, and a car parts store. You also pass a business advertising check cashing, a barber’s shop, a tattoo parlour, a Laudromat, a cheap diner and several boarded up store fronts. The sidewalk is broken up and there are no trees along the street.

*As you walk along the high street a police patrol car drives past. A little later you pass two police officers on foot patrol.*

You turn down a side street. You walk along a street of two storey row houses. Some of the houses are boarded up. The first floor windows have bars on them. There is trash on the ground, particularly as you turn the corner and look down the back alley between the two streets. Walking down the alley you notice barbed wire along the back walls of the houses. Further along there is a garage with a broken window.

*As you exit the street you see a police car drive into the next street along.*

Vignette of affluent neighbourhood with no police presence:

You will now read a short description of a city neighbourhood.

Please read the description carefully.

Imagine you are walking through this neighbourhood.

Walking along the high street you pass a church and some stores. The stores include a delicatessen, an ice-cream parlour, and a ladies clothing store. You also pass a market stall selling fruit and vegetables, a bank, a wellness center, a dry cleaner’s, a restaurant and a hotel. There are trees lining the sidewalk and trash cans along the street.

You turn down a side street. You walk along a street of large townhouses. Most of the houses have planters on their front porches. There are trees lining the street. The cars are parked alongside the kerb.
